# Cancer Stem Cell-Like Cells Derived from Malignant Peripheral Nerve Sheath Tumors

**DOI:** 10.1371/journal.pone.0021099

**Published:** 2011-06-13

**Authors:** Melanie Spyra, Lan Kluwe, Christian Hagel, Rosa Nguyen, Jens Panse, Andreas Kurtz, Victor Felix Mautner, Samuel David Rabkin, Maria Demestre

**Affiliations:** 1 Department of Maxillofacial Surgery, University Medical Center Hamburg-Eppendorf, Hamburg, Germany; 2 Institute of Neuropathology, University Medical Center Hamburg-Eppendorf, Hamburg, Germany; 3 Department of Oncology and Hematology, University Medical Center Hamburg-Eppendorf, Hamburg, Germany; 4 Berlin-Brandenburg Center for Regenerative Therapies (BCRT), Charité Universitätsmedizin Berlin, Berlin, Germany; 5 College of Veterinary Medicine, Seoul National University, Seoul, Republic of Korea; 6 Department of Neurosurgery, Massachusetts General Hospital and Harvard Medical School, Boston, Massachusetts, United States of America; Virginia Commonwealth University, United States of America

## Abstract

This study aims to examine whether or not cancer stem cells exist in malignant peripheral nerve sheath tumors (MPNST). Cells of established lines, primary cultures and freshly dissected tumors were cultured in serum free conditions supplemented with epidermal and fibroblast growth factors. From one established human MPNST cell line, S462, cells meeting the criteria for cancer stem cells were isolated. Clonal spheres were obtained, which could be passaged multiple times. Enrichment of stem cell-like cells in these spheres was also supported by increased expression of stem cell markers such as CD133, Oct4, Nestin and NGFR, and decreased expression of mature cell markers such as CD90 and NCAM. Furthermore, cells of these clonal S462 spheres differentiated into Schwann cells, smooth muscle/fibroblast and neurons-like cells under specific differentiation-inducing cultural conditions. Finally, subcutaneous injection of the spheres into immunodeficient nude mice led to tumor formation at a higher rate compared to the parental adherent cells (66% versus 10% at 2.5×10^5^). These results provide evidence for the existence of cancer stem cell-like cells in malignant peripheral nerve sheath tumors.

## Introduction

Malignant peripheral nerve sheath tumors (MPNSTs) are soft tissue sarcomas arising from peripheral nerves and have high rates of local recurrence and hematogenous metastasis [1]. They account for 10% of all soft tissue sarcomas. Half of these malignancies occur in patients with neurofibromatosis type 1, a tumor suppressor gene syndrome with an incidence of approximately 1 in 3000. MPNST, especially those associated with neurofibromatosis type 1, are one of the most aggressive malignancies in humans with an extremely poor prognosis [2].

Results of recent studies suggest that many malignant tumors contain cells with properties of stem cells including self-renewal, clonality, multipotency, and high rates of tumor formation in immunodeficient mice [3], [4]. Like embryonic stem cells, cancer stem cells are postulated to be drug resistant [5], [6]. In our recent clinical trial using imatinib mesylate, a tyrosine kinase receptor inhibitor, several patients with MPNST showed initially promising responses as their tumors shrunk to very small cores. However, relapse occurred in all cases and the recurring tumors did not respond to the original therapy any more (unpublished observations). Possibly, a small portion of stem cell-like cells escaped the therapy and led to relapse.

While cancer stem cells have been reported and extensively studied in a number of solid tumors [7]–[9], they have not yet been identified in MPNSTs. In the present study, we tested the hypothesis that MPNSTs also contain stem cell-like cells, which can be enriched *in vitro*. The stem cell-like cells from one established human MPNST cell line, S462, were further characterized *in vitro*, *in vivo* and molecularly.

## Materials and Methods

### Patients and tumors

The diagnosis of neurofibromatosis type 1 was made according to the National Institute of Health diagnostic criteria [10]. MPNST tumors (n = 12), plexiform (n = 3) and cutaneous neurofibromas (n = 3) were obtained mostly from the Surgical and Maxillofacial Surgical Departments of the University Medical Center Hamburg-Eppendorf and some from the Neurofibromatosis Clinic, Munich. The obtention of tumors was approved by the local review board (Hamburg Ärztkammer, OB-011/07) and all patients gave written consent. Immediately after surgical removal, the specimens were placed in Hanks buffered saline (Gibco, Paisley, UK). One part of each specimen was used to confirm the pathological diagnosis of the tumor (Institute of Neuropathology, University Hospital Hamburg-Eppendorf). Another part was snap frozen in liquid nitrogen and stored at −80°C. The remaining tissue was used for cell culture.

### Cell culture

Cells of established MPNST cell lines S462 and S1507-2 were cultured under standard conditions with serum [11]. Dissociation and culturing cells from freshly resected MPNSTs were as described previously [11].

### Enrichment of stem cell-like cells

Cells of established lines, primary cultures and freshly dissociated tumors were cultured under stem cell conditions in stem cell medium (SCM) consisting of Neurobasal Medium (Invitrogen, Karlsruhe, Germany) with human recombinant epidermal growth factor (EGF, 20 ng/ml, R&D systems, Minneapolis, MN), basic fibroblast growth factor (bFGF, 20 ng, Prepotech, Rocky Hill, NJ), heparin (32 IE/ml, Rathiopharm, Ulm, Germany) and supplemented with 1% N2 and 1% B27 (both from Invitrogen). Densities of the plated cells ranged from single to 500 cells per well. To passage spheres, spheres were collected by gentle centrifugation at 800 *g* for 5 min, and mechanically dissociated by pipetting up and down using a 1 ml pipette. Thereafter, the cell suspension was filtered through a 30 µm mesh filter to remove remaining aggregates. Single cells were then plated at various densities in SCM.

### Cell proliferation assays

Cell proliferation was assessed using the bromodeoxyuridine incorporation assay (Roche, Mannheim, Germany) following the manufacturer's instructions. Absorbance was read on a spectrophotometer at a wavelength of 405 nm.

### Clonal sphere formation

Cells were plated at low density in 96 well-plates. Wells containing single cells were marked while those containing more than one cell per well were excluded from the analysis. After two (or three) weeks, wells containing spheres were counted. Frequency of sphere formation was calculated as the proportion of wells containing spheres against wells containing single cells.

### Real-time PCR

RNA was prepared from spheres and adherent cells using NucleoSpin RNA XS columns (Macherey Nagel, Düren, Germany). For cDNA synthesis we used random hexamer primers (Fermentas, St.Leon-Rot, Germany) and Revert Aid Reverse Transcriptase (Fermentas). For gene expression analyses validated TaqMan Gene Expression Assays (Applied Biosystems, Darmstadt, Germany) were used. For CD133 discrimination we used TaqMan Gene Expression Assays binding in Exon 2/3 and Exon 4/5 of CD133. Relative amounts of target RNA were normalised to ribosomal protein L13a (RPL13a) as internal control and relative quantitative values were calculated according to the ΔΔCt method.

### Flow cytometry

For detection of surface markers CD133, CD34, nerve growth factor receptor (NGFR, CD271), neural cell adhesion molecule (NCAM, CD56) and CD90, cells were suspended in 30 µl of antibody diluent buffer (Beckman Coulter, Krefeld, Germany) and 10 µl anti-CD133/2-PE (Miltenyi Biotec, Bergisch Gladbach, Germany), 11 µl CD271-FITC (R&D), 5 µl CD34-PE, 5 µl CD56-PC5 or 5 µl CD90-PC7 (Beckmann Coulter) were added (optimal antibody concentrations were determined by several dilution experiments). As negative controls, cells were incubated in the same buffer supplemented with 10 µl IgG1-PE, 10 µl IgG1-FITC, 5 µl IgG1-PC5, 10 µl IgG1-PC7 (Beckmann Coulter) for 30 min at 4°C. For detection of the intracellular antigen Nestin (R&D), cells were fixed and permeabilized with AB reagent following the manufacture's instructions (R&D) and 10 µl Nestin-PE (R&D) or 10 µl IgG1-PE were added for 30 min at 4°C.

After washing with phosphate buffer saline, cells were analyzed within 1 h by flow cytometry either on a FC 500 flow cytometer using CXP software (Beckman Coulter) or on a PAS Particle Analyzing System (Partec, Münster, Germany).

### Magnetic cell sorting

Cells suspended in phosphate buffer saline containing 0.5% Bovine Serum Albumin and 2 mM EDTA were labelled magnetically with anti-CD133/2, CD34 and CD271 microbeads using the Miltenyi Biotec CD133/2, CD34, CD271 microbead kits following the manufacture's instructions. Magnetic separation was carried out with MACS separation columns (Miltenyi Biotec). Positive and negative fractions were eluted three times to increase the purity of the samples. Aliquots of positive and negative sorted cells were evaluated for purity by flow cytometry with a FC 500 flow cytometer using CXP software (Beckman Coulter).

### Cell differentiation assays

Dissociated spheres or parental adherent cells were plated into wells coated with poly-lysine and laminin, and grown in DMEM∶F-12 (3∶1) containing 10% fetal bovine serum. For glia differentiation, 5 ng/ml recombinant human neuregulin-ß1 (kindly provided by Dr. S. Carroll, Division of Neuropathology, Department of Pathology, University of Alabama at Birmingham, Birmingham, Alabama) and 2 mM forskolin (Sigma, Munich, Germany) were added for at least 2 weeks [12]. For smooth muscle fibroblast (SM/Fb) differentiation, 40 ng/ml bFGF (Prepotech) was added for 5 days, and then 1 ng/ml transforming growth factor- ß1 (Sigma) was added for 7 days [12]. For neurons, neurobasal medium was supplemented with 1 mM retinoic acid (Sigma), 10 mg/ml cyclic AMP (Sigma), 1% B27, and 2 mM glutamine [7] for 1 week or longer. For all conditions, medium was changed every three days.

### Immunofluorescence staining

Cells grown on chamber slides were fixed with 4% paraformaldehyde and stained with the following antibodies: rabbit anti-human S100 (1∶500, DAKO, Hamburg, Germany), rabbit anti-smooth muscle actin (1∶100, Santa Cruz, Heidelberg, Germany), mouse anti-human microtubule associated protein (MAP)-2 (Millipore, Schwalbach, Germany), mouse anti-human NGFR (1∶100, Millipore), mouse anti-human neurofilament (1∶400, Millipore) and mouse anti-human Nestin (1∶200, Millipore). For double labelling S100 with either neurofilament or NGFR, the same procedure was used for the second primary antibodies and a second antibody raised in another species and labelled with another dye was used. Appropriate isotype controls and the omission of primary and secondary antibodies were used to rule out possible non-specific immunolabelling. Nuclei were labelled with 4′,6-diamidino-2-phenylindole (Invitrogen).

### Tumorigenicity assay in nude mice

Athymic nude mice (Charles River) were briefly anaesthetised with a mixture of CO_2_/0_2_ and dissociated spheres or adherent cells suspended in 50% Matrigel (R&D) were injected subcutaneously into mice [13]. Tumor growth was monitored twice a week. All animal experiments were approved by the local authority (Behörde für Wissenschaft und Gesundheit, Hamburg, 68/06).

Tumors grown in mice were removed, fixed in 7% formalin and embedded in paraffin. Sections were stained with hematoxylin. For immunohistochemistry we used rabbit anti-human S100 (1∶500) and rabbit anti-human Ki67 (1∶50, Neomarkers, Asbach, Germany) antibodies. For antibody detection, the Envision Kit dual system peroxidase (Dako) was used. Colour reactions were developed with VectorRed (Vector Laboratories, Burlingame, CA) and the nuclei were counterstained with hematoxylin.

### Data analysis

The comparison of frequency of sphere formation between different passages and the increase in the number of cells capable of forming tumor spheres with passaging was tested by a non-parametric ANOVA; if significant, post-hoc comparisons using Dunn's test were performed. The increase in the number of proliferative cells over time was analysed using a non-parametric repeated measurements ANOVA; if significant, post-hoc comparison using Tukeys's test was performed. The same test was performed to compare an increase in proliferation of CD133 positive and negative spheres over time. Differences in the relative amount of cDNA analysed by RT-PCR, and changes in the percentage of positive cells in parental adherent cells and spheres analysed by flow cytometry, were tested using the non-parametric Mann-Whitney U-test. Differences in the frequency of tumor formation between mice injected with adherent cells and spheres respectively, were calculated by the Chi-square test. All averaged values are represented as the mean± standard error of the mean (SEM). Values were considered significant when p<0.05.

## Results

### Sphere formation from MPNST cells

To examine whether or not MPNSTs contain stem-like cells, cells from 2 established lines and 12 dissected tumors were cultured in serum-free medium containing EGF and FGF. Spheres or sphere-like aggregates were obtained from the two cell lines and 10 out of the 12 dissected fresh tumors. Spheres formed at low density, as low as one cell per well, and propagated for at least 20 passages from the established MPNST cell line S462. Spheres or sphere-like aggregates were also obtained from another established MPNST line (S1507-2) and from primary MPNST cells and freshly dissected MPNSTs; however, they survived only for 2 to 12 passages, and could not be obtained at cell densities below 100 cells per well. Similarly, spheres or sphere-like aggregates were also obtained from primary cultures derived from plexiform neurofibromas and cutaneous neurofibromas, but could be not be passaged more than twice. Further experiments thus focused on spheres of S462.

### In vitro characterization of MPNST S462 spheres

**Figure 1:**
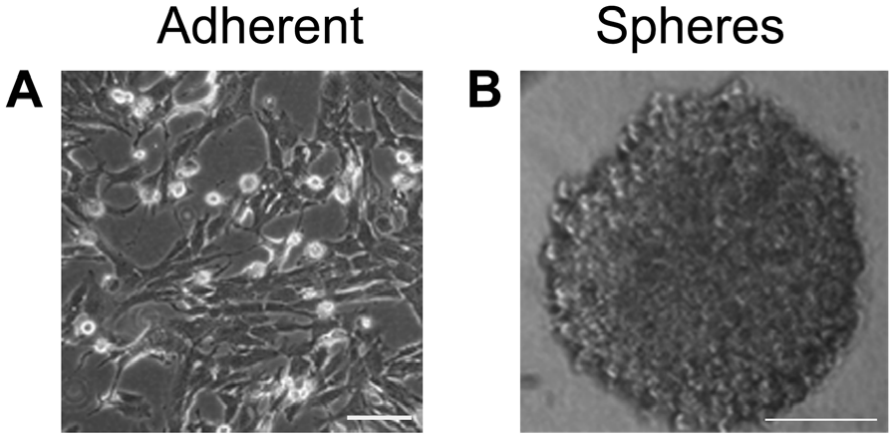
Comparison of MPNST adherent and sphere cultures. (A) Parental S462 cells under standard culture conditions with serum grow adherently (passage 35). (B) Floating secondary sphere after two weeks under stem cell conditions without serum. The sphere was derived from a single cell and thus was clonal. Bars = 20 µm.

**Figure 2:**
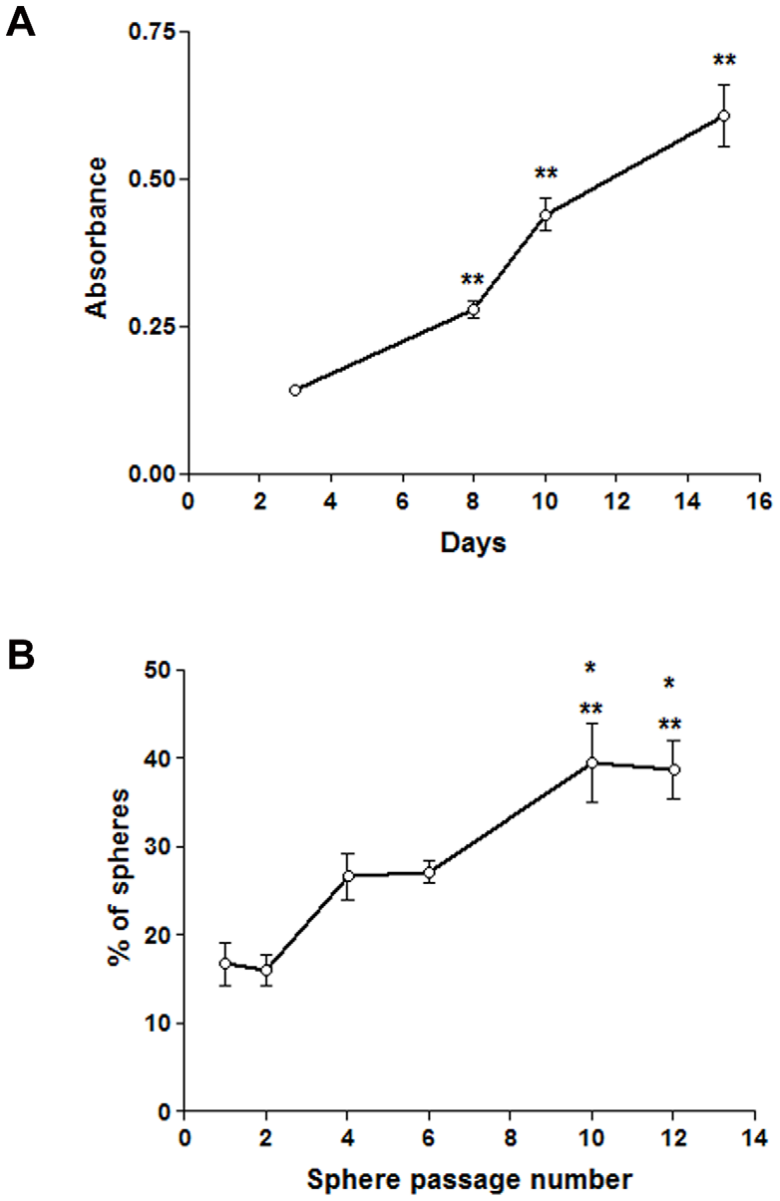
Proliferation and self-renewal of MPNST spheres. Bromodeoxyuridine incorporation assay revealed proliferation of S462 cells in the clonal spheres (secondary spheres). The increase in cell absorbance was significant for 8, 10, and 15 days in SCM versus 3 days (p<0.01 **). Changes in the frequency of sphere formation in wells containing single cells originally from adherent S462, then from dissociated spheres plated as single cells and passaged consecutively. The increase in frequency of sphere formation with increasing passage was significant (passage 1 (primary spheres) versus passages 10 (10^th^ spheres) and 12 (12^th^ spheres), P<0.01 **, passage 2 (secondary spheres) versus passages 10 (10^th^ spheres) and 12^th^ spheres), P<0.05*).

Parental MPNST S462 cells of passage 35, which under standard medium conditions with serum grow adherently, formed floating spheres after 2 days under stem cell conditions (Fig. 1A,B; adherent S462 cells refer to the parental S462 cell line). Proliferation of cells in the spheres demonstrated that these were not just aggregates (Fig. 2A). When these primary spheres were dissociated and the cells plated at extremely low density in wells of a 96-well plate, secondary spheres were obtained in 16.7%±2.4 of wells containing single cells. The frequency of sphere formation from single cells increased when the spheres were passaged further (Fig. 2B). Clonal spheres were also obtained from adherent S462 cells of the very early passage 2, with a higher frequency of primary sphere formation 31.86%±2.50, almost twice as high as with adherent S462 cells of passage 35. To date, MPNST S462 spheres have been passaged under clonal conditions over 20 times.

### Up-regulation of stem cell markers and down regulation of mature cell markers in S462 spheres

**Figure 3:**
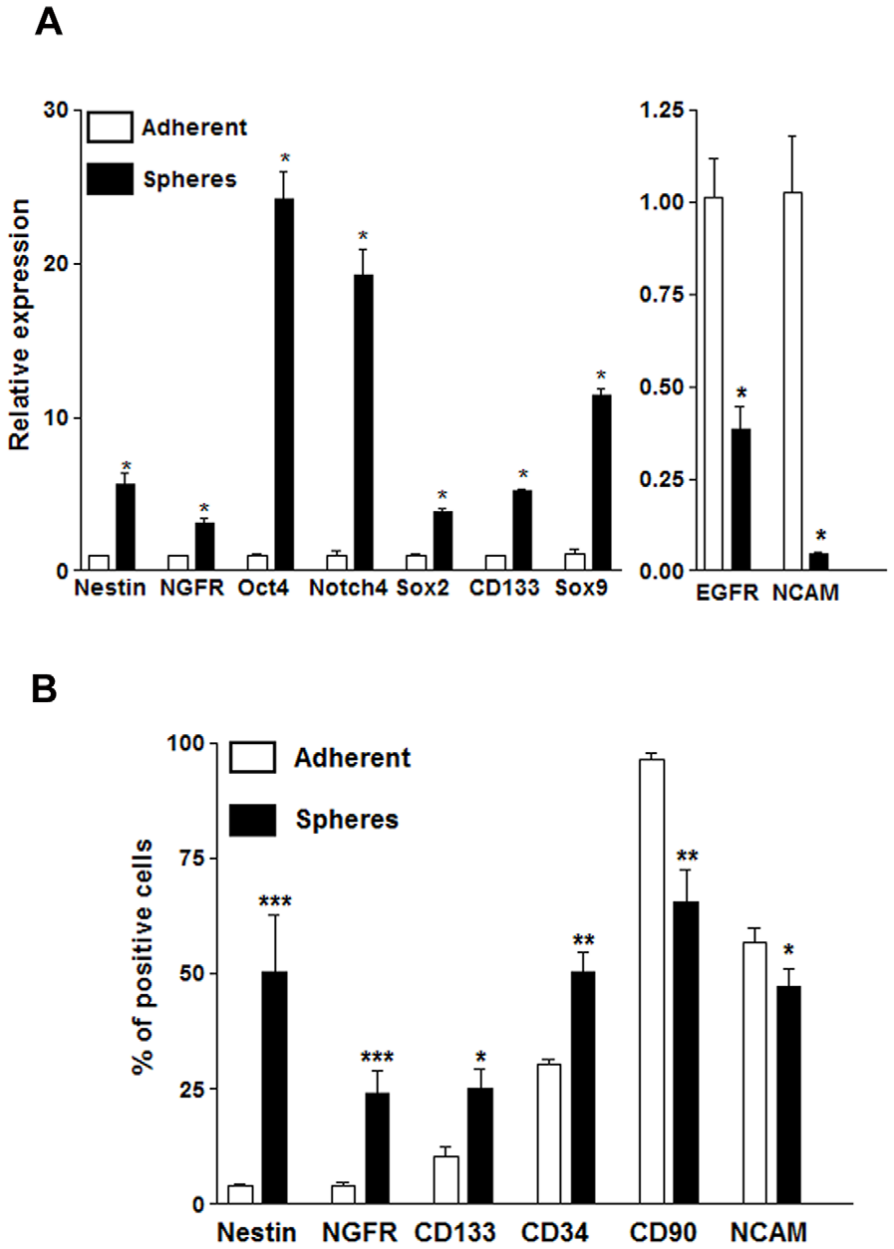
Comparative expression of stem cell markers and mature cell markers in adherent and S462 spheres. (A) Real-Time PCR. Expression of each marker in the spheres was normalized against expression of the same marker in the adherent S462 cells. Spheres at passages between 13 and 16. * p<0.05; adherent versus sphere transcript expression (Nestin, NGFR, Oct4, Sox2, CD133, Sox9, EGFR and NCAM). (B) Flow cytometry. Changes in the percentage of cells expressing mature and stem cell progenitor markers in adherent cells and in spheres (passages 8 and 13), CD133 and NCAM adherent versus spheres p<0.05 *, CD34 and CD90 adherent versus spheres p<0.01 **, NGFR and Nestin adherent versus spheres p<0.01 ***. EGFR, epidermal growth factor receptor; NGFR, nerve growth factor receptor; NCAM, cell adhesion molecule.

Real-Time PCR revealed up-regulation of stem cell markers Nestin, NGFR, Oct4, Notch4, SOX2, CD133 and SOX9 in S462 spheres of passages between 13 and 16, in comparison to S462 adherent cells kept under the SCM culturing conditions for 14–16 hours (Fig. 3A, left). In contrast, EGF receptor (EGFR) and NCAM, a marker of immature Schwann cells, were expressed at lower levels in S462 spheres than in their parental adherent cells (Fig. 3A, right).

**Figure 4:**
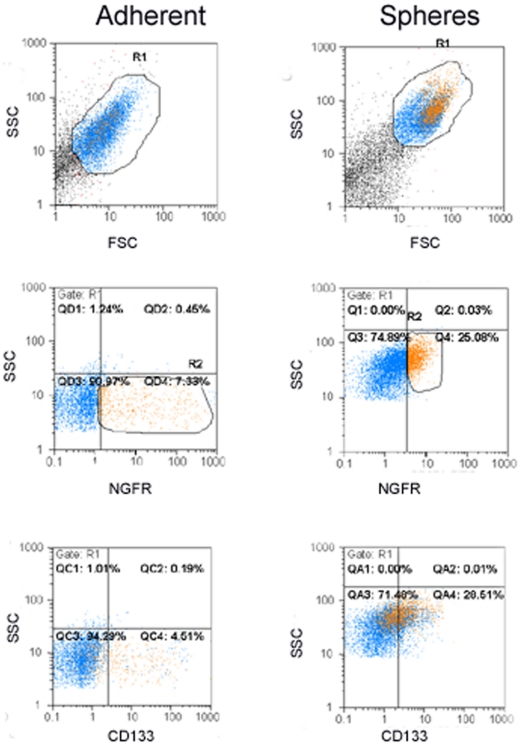
Co-expression of NGFR and CD133 in adherent and S462 spheres. Side and forward scatter analysis of adherent cells (left column) and spheres (at passage 8, right column) showed different types of cells. Live cells were gated in R1 (upper panels), and gatings for NGFR (R2) positive cells (middle panels) are shown in orange. NGFR-positive cells (represented in orange) within the CD133-positive cells show co-expression of CD133/NGFR (lower panels). NGFR cells co-expressing CD133 are infrequent in the adherent cell population and increased in spheres. Isotype controls for all the antigens were used to set up the quadrants for negative populations. NGFR, nerve growth factor receptor.

Flow cytometry revealed increased expression of neural crest markers NGFR, CD133 and Nestin in the spheres compared to parental adherent S462 cells (Fig. 3B). The proportion of cells expressing CD34 was also increased in the spheres to about 50%. In addition, approximately 60% of the NGFR positive population co-expressed-CD133 in adherent S462 and in the spheres (66.84±10.82% adherent versus 60.81±12.21% spheres, not significant), (Fig. 4). Conversely, CD90 and NCAM were expressed less frequently in S462 spheres than in adherent cells (Fig. 3B).

### Higher frequency of S462 sphere formation and proliferation in CD133+ cells

Using magnetic bead-based sorting, we enriched CD133 positive and negative cells from the serum-cultured adherent S462 cells (passage 35) to 90 and 80%, respectively, and cultured them separately in SCM at low cell density. More spheres formed in the CD133+ population when compared to the CD133− population (35.21% versus 18.35%). In addition, cells in CD133+ spheres proliferated more rapidly than those in the CD133− spheres (absorbance at 405 nm of 0.7±0.047 versus 0.39±0.0312, p<0.001).

### S462 spheres are capable of lineage differentiation

To assess the potential for multilineage-differentiation, clonally derived spheres and adherent S462 cells from passage 35 were both cultured in medium containing serum, which induces differentiation. After two to three weeks, expression of S100 was observed in a small number of cells from S462 clonal spheres, indicating lineage differentiation to Schwann cell-like cells. In contrast, expression of this marker was not observed in the adherent S462 cells (data not shown). More than 50% of cells from the clonal spheres expressed Nestin when cultured in serum-containing medium, compared to none of the adherent S462 cells (data not shown).

**Figure 5:**
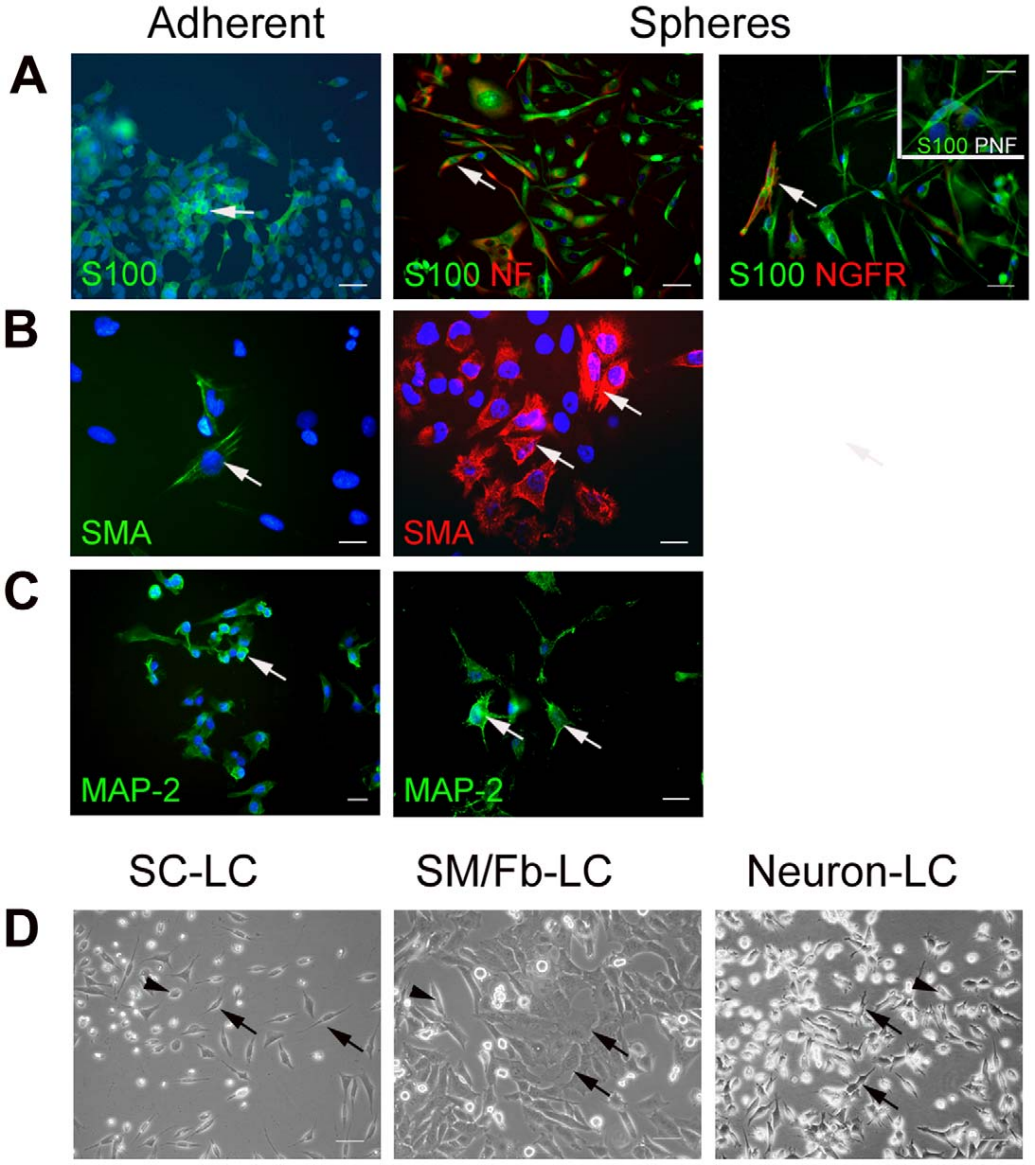
Multi-lineage differentiation of cells of clonal S462 spheres. Adherent cells (left column) and dissociated sphere cells (right columns) were cultured with growth factors inducing differentiation into cells resembling (A) Schwann cells, (B) SM/Fb and (C) neurons, which are positively stained for S100/NGFR/neurofilament (Schwann cells), SMA (SM/Fb), and MAP-2 (neurons), respectively. Insert in A illustrates S100+ Schwann cells derived from a plexiform neurofibroma culture. (D) phase contrast micrographs from differentiated cells under 3 culture conditions to generate Schwann cell, SM/Fb and neuron-like cells. Differentiated cells are indicated by arrows and non-differentiated cells by arrowheads. Bars = 20 µm. NGFR, nerve growth factor; SMA, smooth muscle actin; MAP-2, microtubule associated protein-2; PNF, plexiform neurofibroma; LC, like-cells.

Adding neuregulin and forskolin induced specific differentiation to S100+ Schwann cells in nearly all cells of clonal S462 spheres, but only a small portion of adherent S462 cells were really positive for S100 and showed slight different morphology (Fig. 5A). The bi-polar morphology of the S100+ Schwann cells differentiated from clonal spheres (Fig. 5A, D) was similar to that of Schwann cells derived from a plexiform neurofibroma (Fig. 5A insert). These differentiated cells from clonal spheres also expressed the Schwann cell markers neurofilament and NGFR (Fig. 5A), adherent cells expressed neurofilament but very rarely NGFR (data not shown).

Similarly, exposure to bFGF and transforming growth factor-ß1 induced specific differentiation to SM/Fb-like cells in a large portion of cells from clonal S462 spheres while only a few S462 adherent cells showed smooth muscle actin expression and fibroblastoid morphology (Fig. 5B, D).

Finally, cyclic AMP and retinoic acid induced neurogenic lineage differentiation in cells of clonal spheres, as they expressed MAP-2 and exhibited neuron-specific morphology such as large cell bodies and neurites (Fig. 5C, D). In contrast, while adherent cells expressed MAP-2 under the same cultural conditions (Fig. 5C), these cells did not exhibit neuron-like morphology.

### In vivo characterization of S462 sphere cells

**Figure 6:**
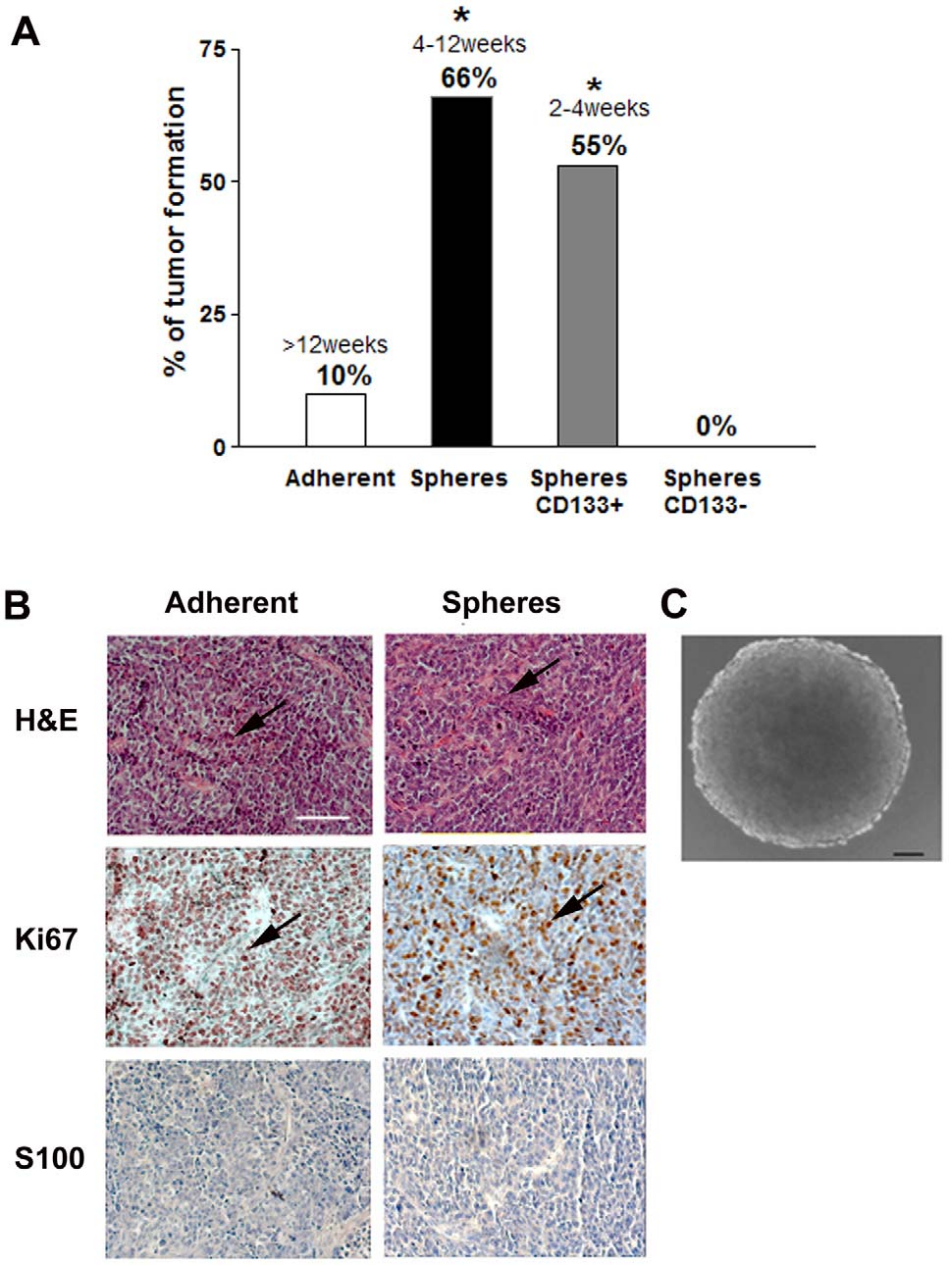
Tumor formation in immunodeficient nude mice. (A) Frequency and time of tumor formation with 2.5×10^5^ adherent (n = 10, passage 35) or clonal sphere S462 (n = 9), CD133^+^ (n = 9) and CD133^−^ (n = 5) cells injected subcutaneously. All sphere cells were obtained between passages 8 and 13. Adherent versus spheres and adherent versus CD133+ spheres p<0.05 *. (B) Histology of tumors derived from adherent cells (left panel) and sphere cells showed that these tumors resemble MPNSTs. H&E shows typical spindle shape cells of MPNST (arrows, top panel), which are highly proliferative by Ki-67 immunstaining (arrows, middle panel) and negative for S100 (bottom panel). Bar = 200 µm, applies for all the micrographs. (C) Cells dissociated from mice tumors formed secondary spheres *in vitro* (representative secondary sphere 3 weeks after plating single cells). Bar = 20 µm.

Subcutaneous injection of 2.5×10^5^ cells of clonal S462 spheres (n = 9) led to visible solid tumor formation 1 to 3 months after injection in 66% of nude mice. In contrast, detectable solid tumors only formed in 10% of nude mice (n = 10) when the same number of adherent S462 cells was injected, this tumor needed over three months to form (Fig. 6A). Histologically, tumors from both adherent cells and spheres exhibited typical characteristics of MPNST: spindle shaped, highly proliferative as shown with high numbers of Ki67 positive cells, and S100 negative tumor cells (Figure 6B).

When 2.5×10^5^ cells from CD133+ spheres (n = 9) and CD133− spheres (n = 5) were injected into nude mice subcutaneously, only CD133+ cells led to visible tumor formation in 55% of the mice, between two weeks and 1 month after injection (Fig. 6A). When 5.6×10^4^ cells from CD133+ spheres (n = 3) were injected, tumors formed in 66% of the mice and took about one month to develop. In contrast, 2.5×10^5^ cells from CD133− spheres did not lead to any visible tumor formation; and higher numbers of cells (5×10^6^, n = 7) led to visible tumors in only 14% of mice and approximately 2 months after injection.

When the tumors from mice were freshly dissociated and plated as single cells in stem cell culture conditions, round spheres appeared within two days (Fig. 6C). These spheres could be grown clonally over consecutive passages. The frequency of sphere formation of cells from tumors grown in mice was approximately 32%, higher than that of adherent S462 cells at passage 35 (which was approximately 17%), while comparable to that of adherent S462 cells at passage 2, which was around 31%.

## Discussion

In this study, we applied culture conditions optimised for neural stem cells, for the enrichment of cancer stem-like cells from MPNSTs. We succeeded in obtaining and enriching such cells from one established MPNST cell line, S462, and thus demonstrated that cancer stem-like cells exist in MPNST. Spheres could be obtained from single MPNST S462 cells, indicating that they are true proliferating spheres and not aggregates of cells. Spheres were also obtained from primary S462 at passage 2, with an even higher frequency. It is thus unlikely that the stem-like cells we obtained, enriched and studied are an artefact of long-term culture.

We could not obtain clonal spheres that could be passaged from a second MPNST cell line, several primary MPNST cultures, freshly dissected tumors or plexiform and cutaneous neurofibromas [12]. MPNST tumors are clinically, genetically and histopathologically very heterogeneous [1]. It is possible that only the S462 line, which was derived from a very aggressive and recurrent tumor, contains an adequate proportion of stem cell-like cells that are immature enough to enable formation of clonal spheres under the selected conditions. Spheres from some other MPNSTs could be passaged at higher densities up to 12 times, indicating some stem cell-like potential. The culture conditions we applied in this study are unlikely to be optimal for cancer stem-like cells in MPNST. This may also be reflected in the difficulties in establishing permanent cultures from fresh tumors, even in standard culture conditions with serum ([14].

S462 cells expressed a number of stem or progenitor cell markers, including Nestin, NFGR CD133 and CD34 to varying degrees, suggesting that these cells are less differentiated and more immature. Expression of these markers was substantially increased in clonal spheres supporting our hypothesis that stem-like cells are enriched in the spheres. CD133+ S462 spheres had a higher frequency of sphere formation *in vitro* and tumor formation in mice when compared to CD133− spheres. This marker thus marks cancer stem-like cells in MPNST to some extent, but not exclusively.

Neural crest stem cells have the ability to self-renew and differentiate into a number of lineages including neurons, glia, and myofibroblasts [15], [16]. In the presence of respective growth factors, cells of the MPNST S462 clonal spheres differentiated into cells resembling Schwann cells, SM/Fb and neurons. Such lineage differentiation was much less profound and less frequent in the adherent MPNST cells. These findings suggest that undifferentiated or dedifferentiated cells, which possess a higher potential for differentiation than differentiated cells, are enriched in clonal S462 spheres. In fact, CD34, a glycophosphoprotein, normally expressed by mature endothelial cells, mesenchymal and hematopoetic progenitor cells, and more importantly in neural stem cells is also localised in MPNSTs tumours, probably in endoneurial fibroblasts [17], [18]. The increase in expression of this marker in the spheres indicates that an increasing number of cells is associated with a more undifferentiated phenotype under the stem cell conditions used. It is also possible that neural stem cell culture conditions induce or promote dedifferentiation of S462 cells or proliferation of stem-like cells present in the parental population.

Self-renewal is one of the properties of stem-like cells. The increase in sphere formation with passaging in stem cell media suggests that the frequency of stem-like cells within the spheres increases with growth, as would be expected if these culture conditions select for, or support increased proliferation, or survival, of stem-like cells. Hence, an enrichment of cancer stem cells in spheres together with reduced proportions of differentiated cells is the likely cause for the increased tumorigenic potency of spheres. Interestingly, as seen in native MPNST, mouse-derived tumors showed indeed no expression of the Schwann cell differentiation marker S100.

Our results provide evidence for the existence of cancer stem or stem cell-like cells in MPNST. These cells grow as clonal spheres for multiple passages under neural stem cell cultural conditions, express stem/progenitor cell markers and induce tumor formation in immunodeficient nude mice at a substantially higher frequency than adherent cells. Studying these cells may contribute to a better understanding of the biology and pathogenesis of MPNST and may enable identification of specific targets for the development of therapies.
